# Altered functional connectivity of the hippocampus in cortico-subcortical networks in early-stage and emerging psychosis

**DOI:** 10.1007/s00406-025-02079-9

**Published:** 2025-08-19

**Authors:** Gina Brunner, Ruchika Gajwani, Joachim Gross, Andrew Gumley, Rajeev Krishnadas, Stephen M. Lawrie, Matthias Schwannauer, Frauke Schultze-Lutter, Peter J. Uhlhaas, Alessio Fracasso

**Affiliations:** 1 School of Psychology and Neuroscience, University of Glasgow, Glasgow, UK; 2 Institute of Health and Wellbeing, University of Glasgow, Glasgow, UK; 3 Institute of Biomagnetism and Biosignalanalysis, University of Muenster, Muenster, Germany; 4 Department of Psychiatry, University of Edinburgh, Edinburgh, UK; 5 Department of Clinical Psychology, University of Edinburgh, Edinburgh, UK; 6 Department of Psychiatry and Psychotherapy, Medical Faculty, Heinrich Heine University, Düsseldorf, Germany; 7 Department of Psychology, Faculty of Psychology, Airlangga University, Airlangga, Indonesia; 8 University Hospital of Child and Adolescent Psychiatry and Psychotherapy, University of Bern, Bern, Switzerland; 9 Department of Child and Adolescent Psychiatry, Charité Universitätsmedizin, Berlin, Germany

**Keywords:** Clinical high-risk, Psychosis, First-episode psychosis, Hippocampus, Brain networks

## Abstract

**Background:**

Deficits in the hippocampus are a consistent finding in schizophrenia and have also been demonstrated in early-stage psychosis. Moreover, alterations in hippocampal anatomy and connectivity have been implicated in aberrant functional interactions in subcortical and cortical networks. However, the nature and extent of these alterations and their association with frontal and subcortical regions remain unclear.

**Methods:**

To address these questions, we analysed resting state fMRI functional connectivity and graph properties in *n* = 93 individuals at clinical high-risk for psychosis (CHR-P), *n* = 26 patients with first-episode psychosis (FEP), *n* = 31 individuals with affective disorders and substance abuse as well as *n* = 58 healthy controls. We used novel denoising techniques and individually optimised functional connectivity matrices, which were compared across clinical groups. Finally, the centrality of the hippocampus as well as network segregation and integration were assessed using graph-based analysis.

**Results:**

Both the FEP and CHR-P groups were characterised by reduced functional connectivity between the hippocampus and inferior frontal cortex albeit the differences in CHR-P individuals did not survive corrections for multiple comparisons. Compared to CHR-P, FEP show lower centrality of the hippocampus but increased network segregation.

**Conclusions:**

Our findings show lower connectivity between the hippocampus and frontal cortex in early-stage psychosis, with FEP patients showing stronger decreases in connectivity compared to CHR-Ps. Furthermore, network-based analyses highlight reduced centrality in FEPs compared to CHR-Ps, indicating reduced influence on the wider network. Thus, altered connectivity along the hippocampal-frontal axis could be a potential marker of illness stage in early-stage psychosis.

**Supplementary Information:**

The online version contains supplementary material available at 10.1007/s00406-025-02079-9.

## Introduction

Schizophrenia (ScZ) is a severe psychiatric disorder which is typically preceded by a prodromal phase, during which attenuated psychotic symptoms, functional deficits and cognitive impairments are present [1, 2]. These observations have led to the formulation of clinical high-risk for psychosis (CHR-P) criteria which are associated with a risk of developing first-episode psychosis (FEP) of approximately 25% over a three-year period [3]. CHR-P participants are characterized by both anatomical and functional brain changes [4–6]. While some such alterations may be stable over time, others may potentially be progressive [7, 8].

The hippocampus is a crucial hub for subcortical and cortical networks and is characterized by extensive anatomical and functional connectivity (FC) with the frontal, occipital and temporal lobes as well as with sensory-motor regions and subcortical areas [9, 10]. Lower hippocampal volumes are already observed in CHR-P participants [11, 12] and may predict transition to psychosis [13] and illness progression [14, 15].

Moreover, the hippocampus supports numerous cognitive functions, such as emotion regulation [16, 17], memory consolidation [18], and social cognition [19]. Several of these domains are impaired in both CHR-Ps [2] and ScZ patients [20]. Decreased task-related activation in combination with elevated baseline BOLD in early-stage psychosis has been demonstrated in the hippocampus [21], which could be due to excessive excitation and may cause volumetric changes [8].

At the circuit level, the hippocampus is involved in regulating striatal dopamine activity. N-methyl-D-aspartate (NMDA) receptor activation causes the nucleus accumbens to inhibit the ventral pallidum via γ-aminobutyric acid (GABA) release, which in turn disinhibits the ventral tegmental area [22, 23]. In animal models of ScZ, hippocampal lesions during early development have been shown to induce disinhibition and behavioural deficits observed in ScZ patients [8]. More specifically, it has been suggested that a loss of GABAergic interneurons and NMDA receptor dysfunction may increase baseline hippocampal excitation [7, 8], resulting in elevated striatal dopamine activity [7].

Excessive baseline excitation may compromise hippocampal [7, 8] and frontal cortex anatomical integrity [24, 25], thus possibly driving more widespread network changes [26], the extent of which may, however, differ across illness stages. Connectivity between the hippocampus and ventral striatum but not frontal regions may be reduced in CHR-P compared to HC, while later stages of illness increasingly involve frontal regions. Particularly chronic ScZ may be associated with global hypoconnectivity [26]. Finally, changes in functional connectivity between subcortical and cortical sites have been shown to predict the development of psychosis in CHR-P individuals [27], highlighting that investigations into large-scale functional networks may be important for the development of biomarkers. Furthermore, differences may exist across illness stages in cortical-subcortical networks [26]. While the hippocampus has a pivotal role in such networks in theoretical and preclinical work [7, 8], few studies have investigated its role in this specific network [28, 29].

The evidence on functional connectivity alterations in extended networks involving the hippocamous in schizophrenia and early-stage psychosis is complemented by evidence from graph theoretical analysis [30, 31]. ScZ patients show altered local but not global network organisation, which may correlate with illness severity [31] (but see [32]). In CHR-P individuals, frontal regions have been shown to operate more in isolation within their local network compared to HC [33–35], while global network properties may be preserved [36].

In the current study, we aimed to advance the understanding of hippocampal connectivity in emerging and early-stage psychosis through the functional connectivity and network-based analysis targeting the hippocampus as well as subcortical and cortical regions in FEP-patients and CHR-Ps. Currently, the precise nature of the aberrant network interactions in early-stage psychosis remains unclear as both higher and lower connectivity patterns have been demonstrated [11, 13, 21]. Lower connectivity between the hippocampus and frontal regions has been demonstrated in FEP, thus we hypothesise that this should also be observed in our sample [28, 29]. Similarly, Sabaroedin and colleagues suggest the CHR-P may be characterised by alterations in connectivity between the hippocampus and subcortex [26]; we would therefore predict differences in hippocampal-subcortical connectivity between CHR-P and HC as well as FEP groups. Another possibility is that the hippocampal-frontal hypoconnectivity identified in FEP is a shared early feature of psychosis, thus we alternatively hypothesise that this may be observed in both CHR-Ps and FEP-patients.

## Methods and materials

### Participants

A total of 289 participants from four clinical groups were included: (1) *n* = 93 participants meeting CHR-P criteria, (2) *n* = 31 participants not meeting CHR-P criteria but who met criteria for affective disorders (*n* = 11), anxiety disorders (*n* = 16), eating disorders (*n* = 1), and/or substance abuse (*n* = 10) (clinical high-risk negative, CHR-N, included as a clinical control group), (3) *n* = 26 FEP patients, and (4) *n* = 58 healthy controls (HC) without an axis I diagnosis or family history of psychosis. Inclusion criteria for FEP-patients were as follows: (1) a DSM-V diagnosis of psychotic disorder and (2) illness duration less than 2 years with only one clinically recorded episode of psychosis. Measurements from a further 18 HC, 17 CHR-P, 7 CHR-N, and 11 FEP were recorded, but excluded due to either missing imaging data, clinically significant incidental findings, or poor image quality.

CHR-P status was established using the Comprehensive Assessment of At Risk Mental States (CAARMS) interview [37] and the Cognitive Disturbances (COGDIS) and Cognitive-Perceptive (COPER) basic symptoms criteria according to the Schizophrenia Proneness Instrument, Adult version [38]. FEP patients were assessed with the Structured Clinical Interview for DSM-5 (SCID) [39] and the Positive and Negative Symptom Scale (PANSS) [40], and were required to have no more than one episode of psychosis, and a duration of illness of less than two years. CHR-P, CHR-N and HC participants were assessed using the Brief Assessment of Cognition in Schizophrenia battery (BACS) [41].

The study was approved by the ethical committees of University of Glasgow and the NHS Research Ethical Committee Glasgow & Greater Clyde. All participants provided written informed consent.

### MRI acquisition

All images were acquired using a Siemens 3T scanner with a 32-channel head coil. T1-weighted images were acquired using a 3D MPRAGE sequence with the following parameters: FoV: 256 × 256 × 176  mm^3^, voxel size: 1 × 1 × 1 mm^3^, TR: 2250ms, TE: 2.6ms, TI: 900ms, FA: 9°. BOLD fMRI was acquired with an EP2D-PACE sequence using the following parameters: FoV: 210mm^3^, voxel size 3 × 3 × 3  mm^3^, TR: 2000ms, TE: 3ms, FA: 77°.

### Neuroimaging processing

**Fig. 1:**
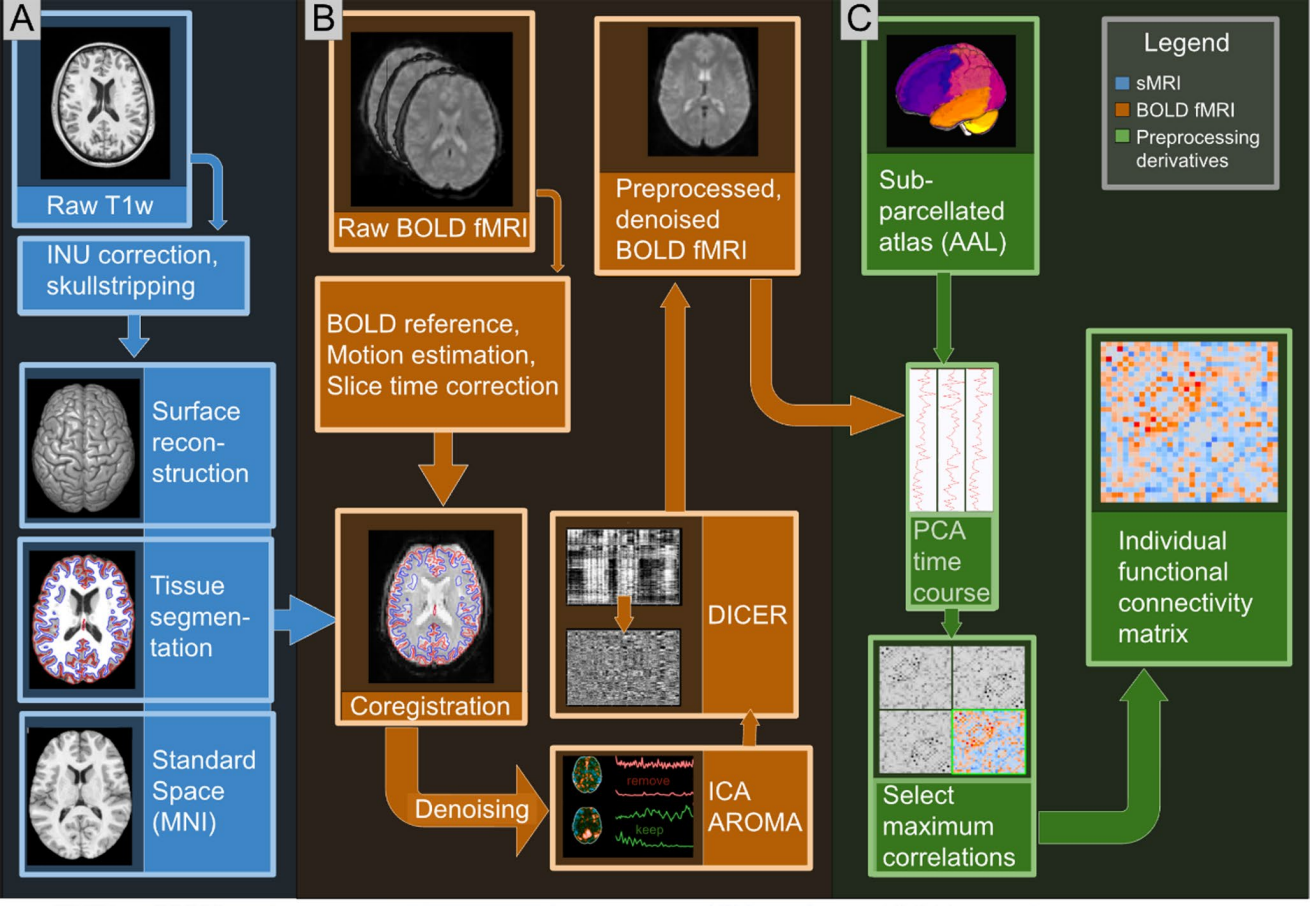
Schematic representation of the preprocessing pipeline. Preprocessing of anatomical (T1-weighted) data is shown in blue (panel **A**), following the standard fmriprep pipeline. BOLD fMRI preprocessing is shown in orange (panel **B**), and includes standard fmriprep preprocessing, as well as additional denoising steps (ICA-AROMA, DiCER). Shown are example topologies and time courses for a noise ICA component (red), and a component which was not rejected (green). The visualisation for DiCER shows two carpet plots, whereby the vertical axis reflects voxel location, and the horizontal axis shows changes in these voxels over time. The upmost carpet plot shows an image pre-DiCER, whereby the ordering of voxels is determined by DBSCAN clustering to visualise large signal deflections. The carpet plot below shows the same image and ordering post-DiCER, whereby the large signal deflections have been removed. Finally, derivatives of preprocessing are shown in green (panel **C**), whereby time courses are extracted with PCA from AAL subparcellated regions (see text below), and the largest correlations per AAL region pair are retained for each subject, thus providing the outputs used for analysis. *sMRI* structural MRI, *T1w* T1-weighted MRI, *fMRI* functional MRI, *AAL* automated anatomical labelling, *DiCER* diffuse cluster estimation and regression, *PCA* principal component analysis

### Fmriprep preprocessing

We used fmriprep 21.0.2 [42] to conduct anatomical and functional data processing. We primarily followed the fmriprep standard pipeline which is described fully in the Methods automatically generated by fmriprep, and can be found in Supplement Methods I. Several options differed from fmriprep defaults: we used 12 degrees of freedom during registration of BOLD to anatomical images, and used ICA-AROMA to reject noise components [43]. ICA-AROMA was chosen due to its ability to retain most data, unlike e.g. censoring approaches with similar denoising performance [44]. ICA-AROMA uses a classifier on FSL MELODIC ICA components to identify and reject noise components, and has previously shown good performance in the removal of motion artifacts in resting state fMRI [44].

Following fmriprep processing, we used ICA-AROMA regressors on the preprocessed BOLD images. In line with prior applications where ICA-AROMA regressors have been applied to data with less or no smoothing [45], the regressors were applied to images smoothed with a 3 mm FWHM Gaussian kernel as a final step.

### Artifact correction

Clinical populations, such as schizophrenia patients, typically show increased head motion which can result in widespread fMRI signal deflections [46, 47]. This can increase local FC while diminishing long range FC [48], and may substantially impact case-control comparisons [44, 49], graph metrics [50], and modelling results [45]. The most common method for removing large signal deflections is global signal regression (GSR), which can remove global signal deflections and thereby strengthen fMRI-behavioural associations [51, 52]. However, GSR has been shown to introduce spurious negative correlations [53], or fail to remove some widespread signal deflections which likely reflect non-neuronal noise [54].

Diffuse Cluster Estimation and Regression (DiCER) is a novel method which uses DBSCAN clustering to identify clusters of widespread signal deflections in fMRI signals, and then using regression to remove it from the signal [54]. DICER attempts at regressing out anomalous clusters rather than the global signal, it is therefore more specific in its denoising than GSR, and captures signal deflections which do not affect all regions equally. As it has further been shown to improve measures of denoising quality such as FC distance dependence [54], we therefore used DiCER to minimise the risk of false positive findings related to artifacts. All preprocessed scans were inspected using ordered carpet plots [54], which allow for the visualisation of widespread signal deflections (see Fig. 1, panel B).

### Functional connectivity extraction

We used the NiPy ecosystem [55] and R to extract time courses and compute functional connectivity matrices. While the AAL1 atlas [56] was used to provide a standardised set of anatomical regions, it has previously been observed that standardised atlases may fail to capture individual variation in functional connectivity [57]. To retain a set of regions comparable across subjects while retaining individual variability, we sub-parcellated each AAL region into 5 subregions using k-means clustering on the AAL atlas image ROI coordinates. We extracted time courses using the 1st principal component from each subregion as PCA has been found to yield more robust FC estimates [58]. Then, z-scored pairwise Pearson’s correlations were computed for between all subregions in ROI pairs, and only the largest correlation (positive or negative) was retained for each pair.

### Functional connectivity analysis

We analysed FC as given by the correlations between the hippocampus and all AAL regions in the medial, lateral, ventral and dorsal frontal cortex, amygdala, caudate, pallidum, putamen, and thalamus, based previous findings [26]. FDR corrected T-tests were used to compare the obtained FC values between ROI pairs from each clinical group to HC. To assess the influence of demographics covariates of FC correlations, we fit a linear model following formula: Correlation ~ Group + Age + Gender, whereby “Correlation” refers to each correlation also tested using t-tests. FDR correction was applied across all resulting linear model outputs (64 models in total).

### Graph analysis

#### Graph generation and edge thresholding

The Python networkx package [59] was used to generate graph representations of individual FC matrices and to extract graph-theoretic metrics. We generated weighted, undirected graphs by using the FC correlation matrices described in the previous step to provide edge weights. Typically, this matrix is then thresholded to provide a sparse representation of network connectivity. While there is no consensus regarding the optimal threshold for graph representations of fMRI data, it has previously been reported that the choice of threshold can significantly impact case-control comparisons, including sign reversals of significant effects [30, 60]. To address these problems, we applied proportional thresholding multiple times, retaining the *n*th percentile from percentiles 1–10 at each step. By using percentiles (per subject), we ensured an equal number of connections for each graph at each step to ascertain that case-control differences will not be introduced by overall differences in FC, but by specific alterations to network function. Finally, absolute FC values were used.

#### Graph metrics

Networkx was used to extract graph metrics for the left and right hippocampus, and local network properties. Based on prior findings in ScZ [31], we focused on local efficiency as well as local clustering (node-level clustering coefficient) and betweenness centrality for the left and right hippocampus. Betweenness centrality was selected as a measure of the ability of the hippocampus to control information flow within the network as it measures the extent to which a given node is on the shortest path connecting other nodes with each other. The local clustering coefficient and local efficiency measure network segregation and integration, respectively. As specified in the Networkx documentation, the former is calculated as *the geometric average of the subgraph edge weights*, while the latter is defined as *the average global efficiency of the subgraph induced by the neighbors of the node* [61].

While metrics are reported at all percentiles to demonstrate the overall effects of thresholding, we restrict statistical testing to the 3rd to 7th percentile. At thresholds below the 3rd percentile, very few edge weights are removed, thus leaving in noisy, small correlations which can distort network properties. Conversely, a substantial proportion of participants’ graph networks became disconnected at thresholds above the 7th percentile, i.e. there was no possible path from each node to every other node. Because the number of included connections can have an impact on the significance and direction of potential effects [30, 60], we inspected whether potential significant effects at different thresholds had the same direction. We averaged the metrics across these percentiles for each participant and compared the clinical groups to HC using a linear model with the formula metric ~ Group. To assess differences at specific thresholds, we conducted t-tests comparing each clinical group to the HC group. FDR-correction was applied across the number of percentiles.

#### Associations with clinical and behavioural variables

ROIs with significant group differences were selected and Pearson’s correlations were computed between FC and PANSS or CAARMS ratings (for FEP and CHR-P, respectively) as well as GAF scores.

## Results

HC and FEP groups were older compared to CHR-Ps, while the FEP group had fewer female participants and lower GAF scores (Table 1).

**Table 1:** Clinical and demographics data

	HC (*N* = 58)	CHR-*N* (*N* = 31)	CHR-*P* (*N* = 93)	FEP (*N* = 26)	Group effect	Post-hoc comparisons
*Age* (M, SD)	24.017 (4.162)	23.19 (4.76)	21.58 (4.26)	24.22 (4.20)	F = 5.12, *p* = .002	HC > CHR-P, FEP > CHR-P
*Gender* (F, %)	40 (58)	18 (58)	67 (72)	10 (38)	X = 11.66, *p* < .01	-
*Medication* (n, %)						
Antidepressant	–	8 (25)	27 (29)	8 (30)	–	–
Antipsychotic	–	0 (0)	0 (0)	13 (50)	–	–
Neither	75 (100)	23 (74)	68 (73)	5 (19)	–	–
*CAARMS severity*						
Total score (M, SD)	–	5.06 (5.85)	32.46 (16.83)	–	t = 9.24, *p* < .001	CHR-P > CHR-N
UTC	–	0.52 (1.12)	2.075 (1.95)	–	–	–
NBI	–	0.74 (1.06)	2.98 (1.78)	–	–	–
PA	–	0.71 (1.27)	2.84 (1.51)	–	–	–
DS	–	0.51 (1.40)	1.51 (1.41)	–	–	
*CHR category*		–				-
CAARMS only (APS/GFRD)	–	–	31	–	–	–
SPI-A only (COGDIS/COPER)	–	–	29	–	–	–
CAARMS + SPI-A	–	–	51	–	–	–
*PANSS severity*						
Total score (M, SD)	–	–	–	55.73 (20.11)	–	–
Positive	–	–	–	13.58 (7.05)	–	–
Negative	–	–	–	10.40 (4.29)	–	–
Cognitive	–	–	–	12.07 (3.33)	–	–
*Global Functioning*				*		
GAF (M, SD)	87.35 (7.15)	70.26 (12.95)	56.68 (11.85)	43.92 (15.45)	F = 68.70, *p* < .001	HC > CHR-N, CHR-P, FEP, CHR-N > CHR-P, FEP, CHR-P > FEP

*APS* attenuated psychotic symptoms, *BACS* Brief Assessment of Cognition in Schizophrenia, *CAARMS* Comprehensive Assessment of At Risk Mental States, *COGDIS* Cognitive Disturbances, *COGDIS* Cognitive-Perceptive Basic Symptoms criterion, *HC* healthy controls, *CHR-N* clinical risk-negative, *CHR-P* clinical high-risk positive, *FEP* first-episode psychosis, *GAF* global assessment of functioning, *SPI-A* Schizophrenia Proneness Instrument, Adult version, *SD* standard deviation of the mean, *AD* antidepressant, *AP* antipsychotic. Note: *Data only available for 12 participants.

### Functional connectivity results

**Fig. 2:**
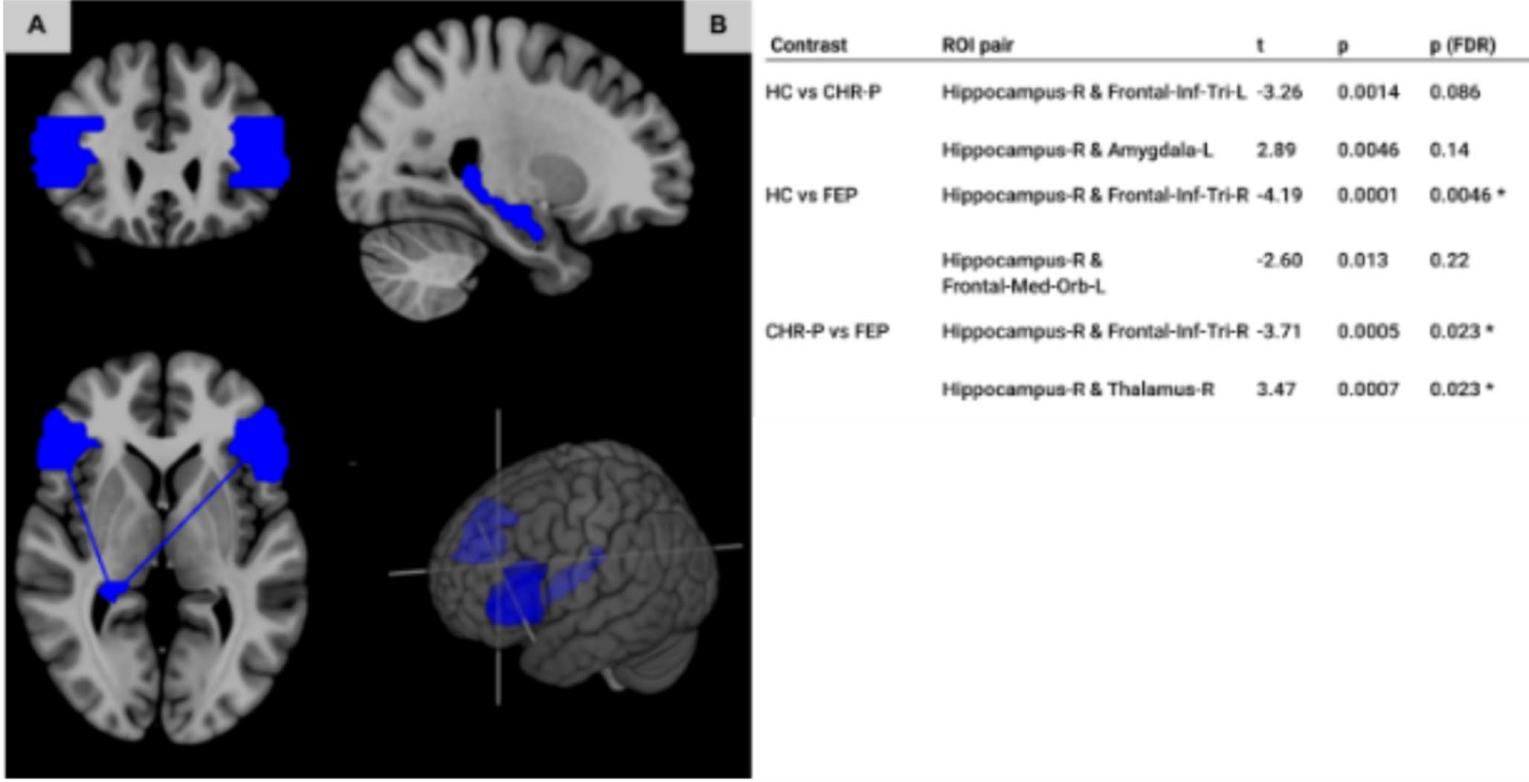
Results from the FC analysis. Panel **A**: Visualisation of loci of FC effects in FEP/CHR-P compared to HC. Shown are the right hippocampus and vlPFC (inferior frontal cortex, pars triangularis, left and right), whereby region masks are based on AAL regions. Regions are visualised in blue to indicate lower FC. Panel **B**: Table showing the two largest effects per group comparison, including corrected (FDR) and uncorrected p-values, rounded to 2 significant figures. Significant (post FDR) effects are highlighted with an asterisk (*). Compared to HC, FEP show lower FC between the hippocampus and vlPFC, which is seen for CHR-P prior to FDR correction only. Compared to CHR-P, FEP show lower FC between hippocampus and vlPFC, and increased FC between hippocampus and thalamus. This figure visualises the results of the t-tests

FEP patients showed lower FC compared to HC between the hippocampus and inferior frontal cortex (pars triangularis, vlPFC) in the right hemisphere (t=-3.41, p_FDR_ =0.028). The CHR-P group showed decreased FC between the right hippocampus and vlPFC (left) compared to HC (t=-3.56, p_FDR_ =0.022), but this was non-significant without correction for age and gender (t=-3.26, p_uncorr_.=0.0014, p_FDR_=0.086). Compared to HC, CHR-Ps had higher FC between the right hippocampus and left amygdala (t = 3.08, p_FDR_ =0.045; however, p_FDR_=0.06 with age and gender covariates). Post-hoc t-tests further revealed a significant difference between the FEP and CHR-P groups indicating lower FC between the right vlPFC and hippocampus in the FEP group (t=-3.71, p_FDR_=0.023, Fig. 2). FC between the right hippocampus and right thalamus was increased in FEP compared to CHR-P (t = 3.47, p_FDR_=0.023). Uncorrected results (p_uncorr_. < 0.02) as well as full results for the CHR-N group can be found in Supplementary Material (see Supplementary Table 1).

### Graph analysis

**Fig. 3:**
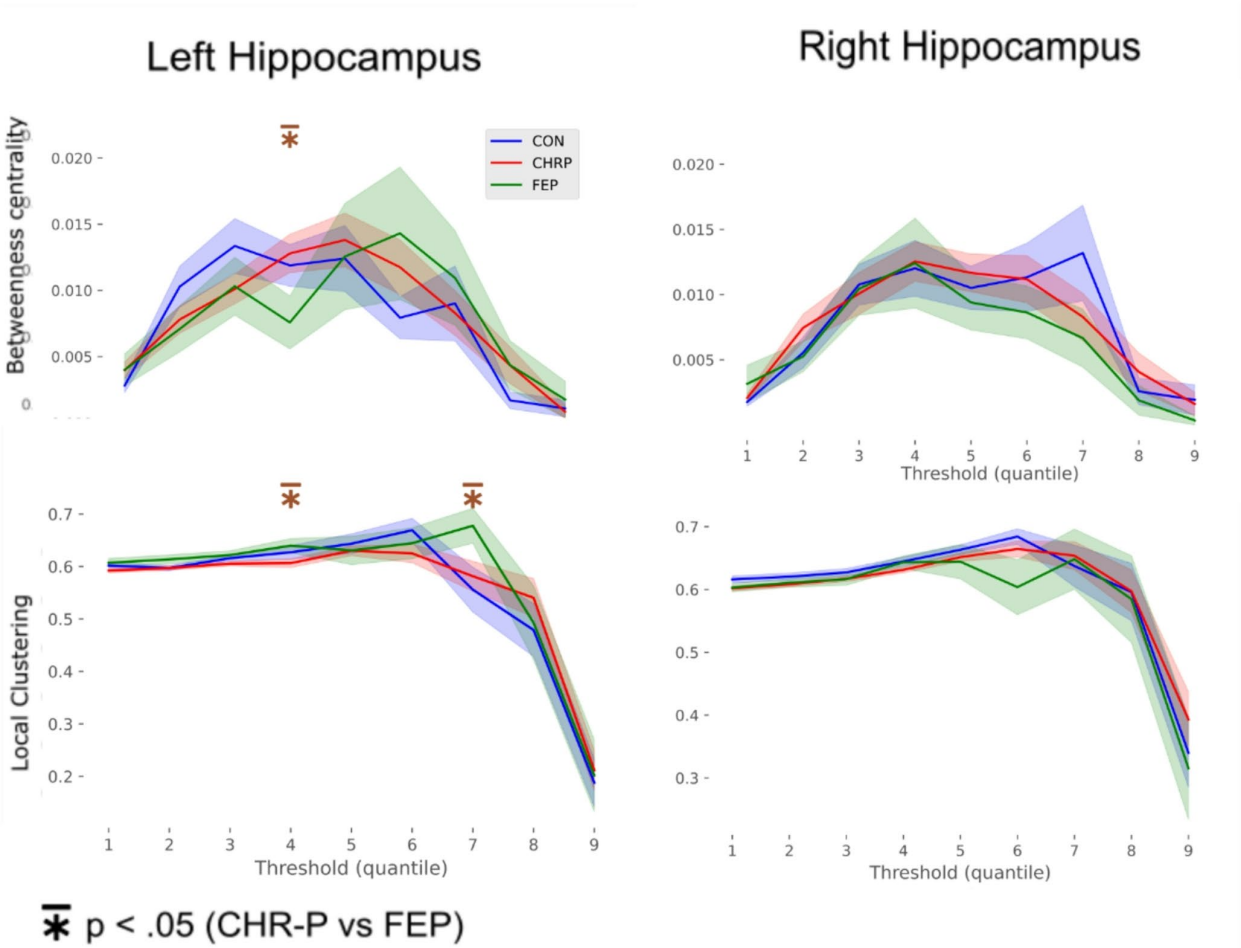
Betweenness centrality and local clustering for the left and right hippocampus. Shown are all thresholding percentiles (1–9), but statistical testing is restricted to percentiles 3–7. Highlighted (*) are significant differences between CHR-P and FEP, with the other groups not showing significant differences; shaded regions reflect one standard deviation from the mean. FEP showed significantly lower hippocampus betweenness centrality compared to CHR-P, and increased local clustering in the left hemisphere. No group differences were detected when averaging across percentiles of interest. Betweenness centrality indicates the extent to which the hippocampus is involved in information flow between different clusters within the network, and local clustering indicates the extent to which the immediate neighbours of the hippocampus are connected to each other

Averaged across the percentiles of interest, no group differences were detected (all *p*s > 0.1). When assessing thresholds individually, FEP patients had significantly lower betweenness centrality of the right hippocampus (t=-4.73, p_corr_=0.0001, 4th percentile), but significantly increased local clustering in the left HP (t = 2.8, p_corr_ =0.0075, 7th percentile; t = 2.19, p_corr_ =0.046, 5th percentile) compared to CHR-Ps (Fig. 3).

### Associations with clinical and behavioural variables

A linear model showed lower right hippocampal volumes in the FEP group (estimate: -203.20, t=-2.074, *p* = .039) compared to controls with total brain volume and age as covariates. The linear model and extraction of volumes with FSL FIRST followed our previous work [11]. Hippocampal volumes were not significantly correlated with FC between right hippocampus and vlPFC (pars triangularis) in either hemisphere, neither betweenness centrality nor local clustering (*p*s > 0.05). Connectivity between the right hippocampus and vlPFC was not associated with betweenness centrality or local clustering (using hemisphere and percentile where group differences were observed). Symptom severity (total PANSS scores and subscales) and functioning (GAF) did not show significant associations with either graph metrics or hippocampal FC with the vlPFC (as described above).

In FEP patients, antipsychotic medication (APM) was associated with higher FC between the right hippocampus and left vlPFC (pars triangularis) compared to those not receiving APM: t = 2.06, *p* = .049. There was no evidence for an effect of antidepressants on FC (hippocampus-vlPFC) or graph metrics (*p* > .05) in CHR-P.

Neither FC between the right hippocampus and vlPFC (pars triangularis, left and right) nor graph metrics were related to symptom severity (CAARMS), functioning (GAF), or right hippocampal volumes in CHR-P (*p*s > 0.05).

## Discussion

We analysed functional connectivity and graph-based network properties in cortical-subcortical networks involving the hippocampus in FEP-patients and CHR-Ps using resting state fMRI. We found evidence for lower functional connectivity between the right hippocampus and vlPFC in FEP patients. Compared to the CHR-P group, FEP patients showed increased connectivity between the right hippocampus and right thalamus. Moreover, APM status was associated with increased FC between the right hippocampus and left vlPFC.

Consistent with previous research, we identified lower FC between the hippocampus and frontal cortex in FEP patients [28, 29, 62, 63]. We did not observe a relationship between PANSS scores and FC however, which indicates that hippocampal-frontal FC is not associated with clinical severity in our sample. Other studies have found hippocampal connectivity to be associated with cognitive function and positive symptoms [28, 29], which is also a prediction of the frameworks which motivated this work [7]. Our relatively small FEP sample size may have limited our ability to detect such associations, although null associations have also been previously reported [64, 65].

Interestingly, the CHR-P group showed lower FC between the hippocampus and inferior frontal cortex as observed in the FEP group. Previous studies identified alterations in the default mode network [66], hippocampal-subcortical FC in a salience processing task [67], as well as hippocampal frontal FC during a memory task [63]. Our findings are broadly consistent with this, but also extend these findings to resting-state fMRI. Moreover, similar alterations in CHR-Ps and FEP patients further suggest that these alterations may already present prior to the onset of psychosis. Mechanistically, this could be related to elevated baseline activity of the hippocampus which has been proposed as an early aetiological factor in the development of psychosis [7, 22].

Although we only identified overlapping alterations in the hippocampus-frontal axis, hippocampal-subcortical connections have previously been implicated in early-stage psychosis also [26]. The majority of our CHR-P cohort did not receive APM which may affect hippocampal-subcortical FC [68]. Similarly, APM status in the FEP group may have affected our ability to detect hippocampal-subcortical alterations, particularly as the sample size is relatively small.

Baseline hyperactivity of the hippocampus may contribute to lower FC in psychosis [69, 70], and this may particularly involve the hippocampal subregion CA1 [71]. To address this possibility, future research may incorporate high-field (7T) fMRI to enable subfield analysis to determine if the reduction in hippocampal-frontal FC identified here can also be linked to CA1.

Moreover, we identified a small association between hippocampal FC and APM-status in FEPs. APM and symptom alleviation has previously been associated with a recovery of hippocampal-striatal functional connectivity in FEPs [65, 68]. This is consistent with the role of the hippocampus in the regulation of dopamine activity [7, 22].

While FEP and CHR-P appear to be characterised by overlapping alterations in hippocampal-frontal FC, graph analysis did not identify a clear origin within the broader network. No group differences in network properties were found when averaging across percentiles, and analysing individual thresholds only revealed differences between the CHR-P and FEP but not with other groups. Lower centrality, as observed in the FEP group at some thresholds, is indicative of lower embedding in the broader network, while increased local clustering suggests stronger connections among immediate neighbours of the hippocampus compared to the CHR-P group [72]. FEP patients have previously shown hypoconnectivity [73], which is reflected anatomically in lower hippocampal structural centrality [74]. Preclinical and theoretical work suggests that impaired hippocampal regulation could induce elevated striatal dopaminergic activity which in turn increases activity in other regions [7, 8, 22]. Our results are only consistent with this to an extent, as they could suggest an effect of illness stage, but not of the presence/absence of psychosis more generally. However, as we did not observe an effect across thresholds, we cannot draw strong inferences from findings at individual percentiles. Consistent with our null finding across percentiles, others have observed mostly intact graph properties in FEP compared to HC, functionally [75] and anatomically [76]. Previous studies have identified lower local network integration in ScZ patients [77], which suggests network alterations may emerge later during illness development.

Another reason for our null finding may be our preprocessing pipeline, which did not use global signal regression and used individualised correlation matrices. The former has been shown to increase potentially spurious negative correlations in patients due to generally lower SNR [53, 54, 78], while the latter has been shown to decrease the number of significant case-control findings in ScZ [79].

In this work, we addressed several limitations typically found in functional connectivity analysis. First, an improved preprocessing pipeline to reduce the impact of noise [44, 54] which tends to be larger in psychosis samples [46–48]. Furthermore, we used individually optimised functional connectivity matrices, which reduce individual variability in functional connectivity estimates [64, 80–82]. Finally, observed alterations in functional connectivity were specific to psychosis and not present in the CHR-N group. This is relevant as reduced network connectivity has been observed in other psychiatric conditions, such as major depressive disorder [83]. However, it should be noted that significantly lower connectivity between the hippocampus and vlPFC was observed in a linear model analysis which used age and gender covariates.

## Limitations

Our FEP sample was relatively small. Moreover, the difference between CHR-P and HC in terms of hippocampal-vlPFC connectivity was no longer significant after correction for multiple comparisons. Moreover, there is currently no methodological consensus regarding graph measures [30] which can limit generalisability (see e.g [32]). Differences between HC and CHR-N were observed following a linear model analysis with age and gender covariates. This is an important limitation of the present work, and future studies should assess whether the specificity of altered hippocampal connectivity to psychosis is robust to correction for demographic covariates. Furthermore, while our subparcellation approach allowed us to take into account individual differences, it does not allow us to draw inferences about anatomical subfields of the hippocampus.

## Conclusion

Resting-state functional connectivity between the hippocampus and frontal cortex is lower in first-episode psychosis. Using novel network-based analyses, our finding implicates the hippocampus as an important factor in altered network organization, suggesting reduced involvement in the information flow between connected cortical and subcortical networks. Thus, altered connectivity along the hippocampal-frontal axis could be a potential biomarker of early-stage psychosis.

To further elucidate the nature and mechanisms of alterations in hippocampal networks in psychosis, future work could investigate the role of neurotransmitter systems in generating these network alterations using MR spectroscopy or PET imaging. This may connect our human findings to past theoretical and preclinical work which highlights an important role for dopaminergic and glutamatergic transmission [7, 8, 22].

## Supplementary Information

Below is the link to the electronic supplementary material.

Supplementary Material 1
