# Supplementary material for: Altered functional connectivity of the hippocampus in cortico-subcortical networks in early-stage and emerging psychosis
Source: Eur Arch Psychiatry Clin Neurosci. 2025 Aug 19;276(3):1053–63. doi: 10.1007/s00406-025-02079-9 (PMC13002657; doi:10.1007/s00406-025-02079-9)
Supplement: Supplementary file 1 — Supplementary Material 1 [file 406_2025_2079_MOESM1_ESM.docx]

Supplementary Information

Supplement I: fmriprep preprocessing

We used the fmriprep package (21 series) with the following command: /opt/conda/bin/fmriprep /data /out participant --use-aroma --skip-bids-validation --bold2t1w-dof 12 --output-spaces MNI152NLin2009cAsym. The following description is automatically provided by fmriprep and is intended to be provided unaltered; please note that it refers to one subject due to the command being run in a loop, but the same processing was applied to the entire dataset. No changes were made to the below text except for formatting adjustments:

Results included in this manuscript come from preprocessing performed using *fMRIPrep* 21.0.2 (Esteban, Markiewicz, et al. (2018); Esteban, Blair, et al. (2018); RRID:SCR_016216), which is based on *Nipype* 1.6.1 (K. Gorgolewski et al. (2011); K. J. Gorgolewski et al. (2018); RRID:SCR_002502).

**Preprocessing of B_0_ inhomogeneity mappings**

A total of 1 fieldmaps were found available within the input BIDS structure for this particular subject. A *B_0_* nonuniformity map (or *fieldmap*) was estimated from the phase-drift map(s) measure with two consecutive GRE (gradient-recalled echo) acquisitions. The corresponding phase-map(s) were phase-unwrapped with prelude (FSL 6.0.5.1:57b01774).

**Anatomical data preprocessing**

A total of 1 T1-weighted (T1w) images were found within the input BIDS dataset. The T1-weighted (T1w) image was corrected for intensity non-uniformity (INU) with N4BiasFieldCorrection (Tustison et al. 2010), distributed with ANTs 2.3.3 (Avants et al. 2008, RRID:SCR_004757), and used as T1w-reference throughout the workflow. The T1w-reference was then skull-stripped with a *Nipype* implementation of the antsBrainExtraction.sh workflow (from ANTs), using OASIS30ANTs as target template. Brain tissue segmentation of cerebrospinal fluid (CSF), white-matter (WM) and gray-matter (GM) was performed on the brain-extracted T1w using fast (FSL 6.0.5.1:57b01774, RRID:SCR_002823, Zhang, Brady, and Smith 2001). Brain surfaces were reconstructed using recon-all (FreeSurfer 6.0.1, RRID:SCR_001847, Dale, Fischl, and Sereno 1999), and the brain mask estimated previously was refined with a custom variation of the method to reconcile ANTs-derived and FreeSurfer-derived segmentations of the cortical gray-matter of Mindboggle (RRID:SCR_002438, Klein et al. 2017). Volume-based spatial normalization to two standard spaces (MNI152NLin2009cAsym, MNI152NLin6Asym) was performed through nonlinear registration with antsRegistration (ANTs 2.3.3), using brain-extracted versions of both T1w reference and the T1w template. The following templates were selected for spatial normalization: *ICBM 152 Nonlinear Asymmetrical template version 2009c* [Fonov et al. (2009), RRID:SCR_008796; TemplateFlow ID: MNI152NLin2009cAsym], *FSL’s MNI ICBM 152 non-linear 6th Generation Asymmetric Average Brain Stereotaxic Registration Model* [Evans et al. (2012), RRID:SCR_002823; TemplateFlow ID: MNI152NLin6Asym].

**Functional data preprocessing**

For each of the 1 BOLD runs found per subject (across all tasks and sessions), the following preprocessing was performed. First, a reference volume and its skull-stripped version were generated using a custom methodology of *fMRIPrep*. Head-motion parameters with respect to the BOLD reference (transformation matrices, and six corresponding rotation and translation parameters) are estimated before any spatiotemporal filtering using mcflirt (FSL 6.0.5.1:57b01774, Jenkinson et al. 2002). BOLD runs were slice-time corrected to 0.962s (0.5 of slice acquisition range 0s-1.93s) using 3dTshift from AFNI (Cox and Hyde 1997, RRID:SCR_005927). The BOLD time-series (including slice-timing correction when applied) were resampled onto their original, native space by applying the transforms to correct for head-motion. These resampled BOLD time-series will be referred to as *preprocessed BOLD in original space*, or just *preprocessed BOLD*. The BOLD reference was then co-registered to the T1w reference using bbregister (FreeSurfer) which implements boundary-based registration (Greve and Fischl 2009). Co-registration was configured with twelve degrees of freedom to account for distortions remaining in the BOLD reference. Several confounding time-series were calculated based on the *preprocessed BOLD*: framewise displacement (FD), DVARS and three region-wise global signals. FD was computed using two formulations following Power (absolute sum of relative motions, Power et al. (2014)) and Jenkinson (relative root mean square displacement between affines, Jenkinson et al. (2002)). FD and DVARS are calculated for each functional run, both using their implementations in *Nipype* (following the definitions by Power et al. 2014). The three global signals are extracted within the CSF, the WM, and the whole-brain masks. Additionally, a set of physiological regressors were extracted to allow for component-based noise correction (*CompCor*, Behzadi et al. 2007). Principal components are estimated after high-pass filtering the *preprocessed BOLD* time-series (using a discrete cosine filter with 128s cut-off) for the two *CompCor* variants: temporal (tCompCor) and anatomical (aCompCor). tCompCor components are then calculated from the top 2% variable voxels within the brain mask. For aCompCor, three probabilistic masks (CSF, WM and combined CSF+WM) are generated in anatomical space. The implementation differs from that of Behzadi et al. in that instead of eroding the masks by 2 pixels on BOLD space, the aCompCor masks are subtracted a mask of pixels that likely contain a volume fraction of GM. This mask is obtained by dilating a GM mask extracted from the FreeSurfer’s *aseg* segmentation, and it ensures components are not extracted from voxels containing a minimal fraction of GM. Finally, these masks are resampled into BOLD space and binarized by thresholding at 0.99 (as in the original implementation). Components are also calculated separately within the WM and CSF masks. For each CompCor decomposition, the *k* components with the largest singular values are retained, such that the retained components’ time series are sufficient to explain 50 percent of variance across the nuisance mask (CSF, WM, combined, or temporal). The remaining components are dropped from consideration. The head-motion estimates calculated in the correction step were also placed within the corresponding confounds file. The confound time series derived from head motion estimates and global signals were expanded with the inclusion of temporal derivatives and quadratic terms for each (Satterthwaite et al. 2013). Frames that exceeded a threshold of 0.5 mm FD or 1.5 standardised DVARS were annotated as motion outliers. The BOLD time-series were resampled into standard space, generating a *preprocessed BOLD run in MNI152NLin2009cAsym space*. First, a reference volume and its skull-stripped version were generated using a custom methodology of *fMRIPrep*. Automatic removal of motion artifacts using independent component analysis (ICA-AROMA, Pruim et al. 2015) was performed on the *preprocessed BOLD on MNI space* time-series after removal of non-steady state volumes and spatial smoothing with an isotropic, Gaussian kernel of 6mm FWHM (full-width half-maximum). Corresponding “non-aggresively” denoised runs were produced after such smoothing. Additionally, the “aggressive” noise-regressors were collected and placed in the corresponding confounds file. All resamplings can be performed with *a single interpolation step* by composing all the pertinent transformations (i.e. head-motion transform matrices, susceptibility distortion correction when available, and co-registrations to anatomical and output spaces). Gridded (volumetric) resamplings were performed using antsApplyTransforms (ANTs), configured with Lanczos interpolation to minimize the smoothing effects of other kernels (Lanczos 1964). Non-gridded (surface) resamplings were performed using mri_vol2surf (FreeSurfer).

Many internal operations of *fMRIPrep* use *Nilearn* 0.8.1 (Abraham et al. 2014, RRID:SCR_001362), mostly within the functional processing workflow. For more details of the pipeline, see [the section corresponding to workflows in *fMRIPrep*’s documentation](https://fmriprep.readthedocs.io/en/latest/workflows.html).

Copyright Waiver

The above boilerplate text was automatically generated by fMRIPrep with the express intention that users should copy and paste this text into their manuscripts *unchanged*. It is released under the [CC0](https://creativecommons.org/publicdomain/zero/1.0/) license.

Note that the fmriprep pipeline was run exactly as described, but in line with prior applications where ICA-AROMA regressors have been applied to data with less or no smoothing (Aquino et al., 2022), the regressors were applied to images smoothed with a 3mm FWHM Gaussian kernel as a final step. ICA-AROMA was chosen due to its ability to retain most data, unlike e.g. censoring approaches with similar denoising performance (Parkes, Fulcher, Yücel, & Fornito, 2018).

References

Abraham, Alexandre, Fabian Pedregosa, Michael Eickenberg, Philippe Gervais, Andreas Mueller, Jean Kossaifi, Alexandre Gramfort, Bertrand Thirion, and Gael Varoquaux. 2014. “Machine Learning for Neuroimaging with Scikit-Learn.” *Frontiers in Neuroinformatics* 8. <https://doi.org/10.3389/fninf.2014.00014>.

Avants, B. B., C. L. Epstein, M. Grossman, and J. C. Gee. 2008. “Symmetric Diffeomorphic Image Registration with Cross-Correlation: Evaluating Automated Labeling of Elderly and Neurodegenerative Brain.” *Medical Image Analysis* 12 (1): 26–41. <https://doi.org/10.1016/j.media.2007.06.004>.

Behzadi, Yashar, Khaled Restom, Joy Liau, and Thomas T. Liu. 2007. “A Component Based Noise Correction Method (CompCor) for BOLD and Perfusion Based fMRI.” *NeuroImage* 37 (1): 90–101. <https://doi.org/10.1016/j.neuroimage.2007.04.042>.

Cox, Robert W., and James S. Hyde. 1997. “Software Tools for Analysis and Visualization of fMRI Data.” *NMR in Biomedicine* 10 (4-5): 171–78. [https://doi.org/10.1002/(SICI)1099-1492(199706/08)10:4/5<171::AID-NBM453>3.0.CO;2-L](https://doi.org/10.1002/(SICI)1099-1492(199706/08)10:4/5%3C171::AID-NBM453%3E3.0.CO;2-L).

Dale, Anders M., Bruce Fischl, and Martin I. Sereno. 1999. “Cortical Surface-Based Analysis: I. Segmentation and Surface Reconstruction.” *NeuroImage* 9 (2): 179–94. <https://doi.org/10.1006/nimg.1998.0395>.

Esteban, Oscar, Ross Blair, Christopher J. Markiewicz, Shoshana L. Berleant, Craig Moodie, Feilong Ma, Ayse Ilkay Isik, et al. 2018. “fMRIPrep.” *Software*. <https://doi.org/10.5281/zenodo.852659>.

Esteban, Oscar, Christopher Markiewicz, Ross W Blair, Craig Moodie, Ayse Ilkay Isik, Asier Erramuzpe Aliaga, James Kent, et al. 2018. “fMRIPrep: A Robust Preprocessing Pipeline for Functional MRI.” *Nature Methods*. <https://doi.org/10.1038/s41592-018-0235-4>.

Evans, AC, AL Janke, DL Collins, and S Baillet. 2012. “Brain Templates and Atlases.” *NeuroImage* 62 (2): 911–22. <https://doi.org/10.1016/j.neuroimage.2012.01.024>.

Fonov, VS, AC Evans, RC McKinstry, CR Almli, and DL Collins. 2009. “Unbiased Nonlinear Average Age-Appropriate Brain Templates from Birth to Adulthood.” *NeuroImage* 47, Supplement 1: S102. <https://doi.org/10.1016/S1053-8119(09)70884-5>.

Gorgolewski, K., C. D. Burns, C. Madison, D. Clark, Y. O. Halchenko, M. L. Waskom, and S. Ghosh. 2011. “Nipype: A Flexible, Lightweight and Extensible Neuroimaging Data Processing Framework in Python.” *Frontiers in Neuroinformatics* 5: 13. <https://doi.org/10.3389/fninf.2011.00013>.

Gorgolewski, Krzysztof J., Oscar Esteban, Christopher J. Markiewicz, Erik Ziegler, David Gage Ellis, Michael Philipp Notter, Dorota Jarecka, et al. 2018. “Nipype.” *Software*. <https://doi.org/10.5281/zenodo.596855>.

Greve, Douglas N, and Bruce Fischl. 2009. “Accurate and Robust Brain Image Alignment Using Boundary-Based Registration.” *NeuroImage* 48 (1): 63–72. <https://doi.org/10.1016/j.neuroimage.2009.06.060>.

Jenkinson, Mark, Peter Bannister, Michael Brady, and Stephen Smith. 2002. “Improved Optimization for the Robust and Accurate Linear Registration and Motion Correction of Brain Images.” *NeuroImage* 17 (2): 825–41. <https://doi.org/10.1006/nimg.2002.1132>.

Klein, Arno, Satrajit S. Ghosh, Forrest S. Bao, Joachim Giard, Yrjö Häme, Eliezer Stavsky, Noah Lee, et al. 2017. “Mindboggling Morphometry of Human Brains.” *PLOS Computational Biology* 13 (2): e1005350. <https://doi.org/10.1371/journal.pcbi.1005350>.

Lanczos, C. 1964. “Evaluation of Noisy Data.” *Journal of the Society for Industrial and Applied Mathematics Series B Numerical Analysis* 1 (1): 76–85. <https://doi.org/10.1137/0701007>.

Power, Jonathan D., Anish Mitra, Timothy O. Laumann, Abraham Z. Snyder, Bradley L. Schlaggar, and Steven E. Petersen. 2014. “Methods to Detect, Characterize, and Remove Motion Artifact in Resting State fMRI.” *NeuroImage* 84 (Supplement C): 320–41. <https://doi.org/10.1016/j.neuroimage.2013.08.048>.

Pruim, Raimon H. R., Maarten Mennes, Daan van Rooij, Alberto Llera, Jan K. Buitelaar, and Christian F. Beckmann. 2015. “ICA-AROMA: A Robust ICA-Based Strategy for Removing Motion Artifacts from fMRI Data.” *NeuroImage* 112 (Supplement C): 267–77. <https://doi.org/10.1016/j.neuroimage.2015.02.064>.

Satterthwaite, Theodore D., Mark A. Elliott, Raphael T. Gerraty, Kosha Ruparel, James Loughead, Monica E. Calkins, Simon B. Eickhoff, et al. 2013. “An improved framework for confound regression and filtering for control of motion artifact in the preprocessing of resting-state functional connectivity data.” *NeuroImage* 64 (1): 240–56. <https://doi.org/10.1016/j.neuroimage.2012.08.052>.

Tustison, N. J., B. B. Avants, P. A. Cook, Y. Zheng, A. Egan, P. A. Yushkevich, and J. C. Gee. 2010. “N4itk: Improved N3 Bias Correction.” *IEEE Transactions on Medical Imaging* 29 (6): 1310–20. <https://doi.org/10.1109/TMI.2010.2046908>.

Zhang, Y., M. Brady, and S. Smith. 2001. “Segmentation of Brain MR Images Through a Hidden Markov Random Field Model and the Expectation-Maximization Algorithm.” *IEEE Transactions on Medical Imaging* 20 (1): 45–57. <https://doi.org/10.1109/42.906424>.

Aquino, K. M., Fulcher, B., Oldham, S., Parkes, L., Gollo, L., Deco, G., & Fornito, A. (2022). On the intersection between data quality and dynamical modelling of large-scale fMRI signals. *Neuroimage, 256*, 119051.

Parkes, L., Fulcher, B., Yücel, M., & Fornito, A. (2018). An evaluation of the efficacy, reliability, and sensitivity of motion correction strategies for resting-state functional MRI. *Neuroimage, 171*, 415-436.

Supplementary Table 1: Functional connectivity analysis, all results where p < 0.02 (before rounding). Significant results (post-FDR correction) are highlighted with *.

| **Contrast** | **ROI pair** | **t** | **p** | **p (FDR)** |
| --- | --- | --- | --- | --- |
| HC vs CHR-P | Hippocampus-R & Frontal-Inf-Tri-L | -3.26 | 0.0014 | 0.086 |
|  | Hippocampus-R & Amygdala-L | 2.89 | 0.0046 | 0.14 |
|  | Hippocampus-R & Putamen-R | -2.75 | 0.0067 | 0.14 |
|  | Hippocampus-L & Caudate-R | 2.51 | 0.013 | 0.21 |
| HC vs FEP | Hippocampus-R & Frontal-Inf-Tri-R | -4.19 | 0.0001 | 0.0046 * |
|  | Hippocampus-R & Frontal-Med-Orb-L | -2.60 | 0.013 | 0.22 |
| CHR-P vs FEP | Hippocampus-R & Frontal-Inf-Tri-R | -3.71 | 0.0005 | 0.023 * |
|  | Hippocampus-R & Thalamus-R | 3.47 | 0.0007 | 0.023 * |
|  | Hippocampus-R & Frontal-Sup-R | -2.55 | 0.0129 | 0.27 |
| HC vs CHR-N | Hippocampus-R & Frontal-Inf-Tri-L | 3.45 | 0.0014 | 0.054 |
|  | Hippocampus-R & Putamen-R | 3.34 | 0.0020 | 0.054 |
|  | Hippocampus-R & Pallidum-L | 3.16 | 0.0026 | 0.054 |
| CHR-N vs FEP | Hippocampus-R & Frontal-Inf-Tri-R | -3.094 | 0.003 | 0.20 |
|  | Hippocampus-R & Frontal-Sup-R | -2.509 | 0.016 | 0.33 |
| CHR-N vs FEP | Hippocampus-L & Frontal-Sup-Orb-R | 2.482 | 0.016 | 0.33 |
|  | Hippocampus-R & Frontal-Inf-Tri-R | -3.094 | 0.003 | 0.20 |
| CHR-N vs CHR-P | Hippocampus-L & Caudate-L | 3.06 | 0.0032 | 0.18 |
|  | Hippocampus-R & Pallidum-L | -2.82 | 0.0072 | 0.18 |
|  | Hippocampus-L & Caudate-R | -2.72 | 0.0089 | 0.18 |

Supplementary Table 2: Functional connectivity analysis with linear models using age and gender covariates. All group differences are relative to HC.

| **Group** | **ROI pair** | **t** | **p** | **p (FDR)** |
| --- | --- | --- | --- | --- |
| CHR-N | Hippocampus-R & Frontal-Inf-Tri-L | -3.91 | 0.00013 | 0.0121 |
|  | Putamen-R & Hippocampus-R | -3.84 | 0.00017 | 0.0124 |
|  | Pallidum-L & Hippocampus-R | -3.52 | 0.00055 | 0.0229 |
|  | Hippocampus-R & Frontal-Inf-Oper-L | -2.496 | 0.013 | 0.2030 |
|  | Hippocampus-L & Hippocampus-R | -2.403 | 0.017 | 0.2104 |
|  | Hippocampus-R & Hippocampus-L | -2.403 | 0.017 | 0.2104 |
|  | Hippocampus-L & Frontal-Sup-Orb-R | -2.387 | 0.018 | 0.2127 |
| CHR-P | Hippocampus-R & Frontal-Inf-Tri-L | -3.56 | 0.00048 | 0.0225 |
|  | Amygdala-L & Hippocampus-R | 3.076 | 0.002 | 0.0625 |
|  | Caudate-R & Hippocampus-L | 2.861 | 0.005 | 0.1048 |
|  | Hippocampus-L & Frontal-Inf-Orb-R | -2.765 | 0.006 | 0.1226 |
|  | Thalamus-R & Hippocampus-R | -2.726 | 0.007 | 0.1266 |
|  | Hippocampus-R & Frontal-Mid-Orb-L | 2.606 | 0.010 | 0.1623 |
|  | Hippocampus-L & Frontal-Mid-Orb-R | -2.423 | 0.016 | 0.2104 |
| FEP | Hippocampus-R & Frontal-Inf-Tri-R | -3.41 | 0.00081 | 0.0279 |
|  | Hippocampus-L & Frontal-Mid-Orb-L | -2.647 | 0.009 | 0.1518 |

Supplementary figures 1-2: FC analysis results including CHR-N


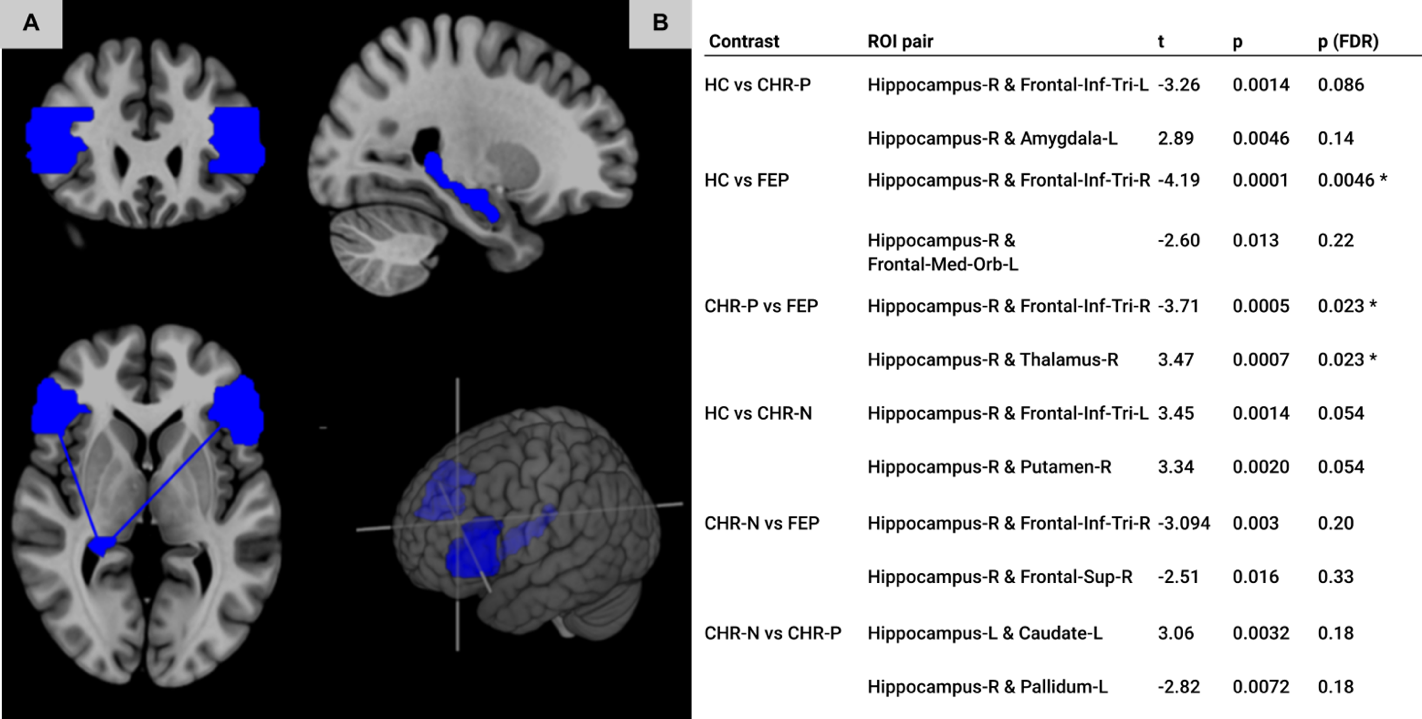


**Supp. Figure 1**.: Results from the FC analysis. Panel A: Visualisation of loci of FC effects in FEP/CHR-P compared to HC. Shown are the right hippocampus and vlPFC (inferior frontal cortex, pars triangularis, left and right), whereby region masks are based on AAL regions. Regions are visualised in blue to indicate lower FC. Panel B: Table showing the two largest effects per group comparison, including corrected (FDR) and uncorrected p-values, rounded to 2 significant figures. Significant (post FDR) effects are highlighted with an asterisk (*). Compared to HC, FEP show lower FC between the hippocampus and vlPFC, which is seen for CHR-P prior to FDR correction only. Compared to CHR-P, FEP show lower FC between hippocampus and vlPFC, and increased FC between hippocampus and thalamus. This figure visualises the results of the t-tests.


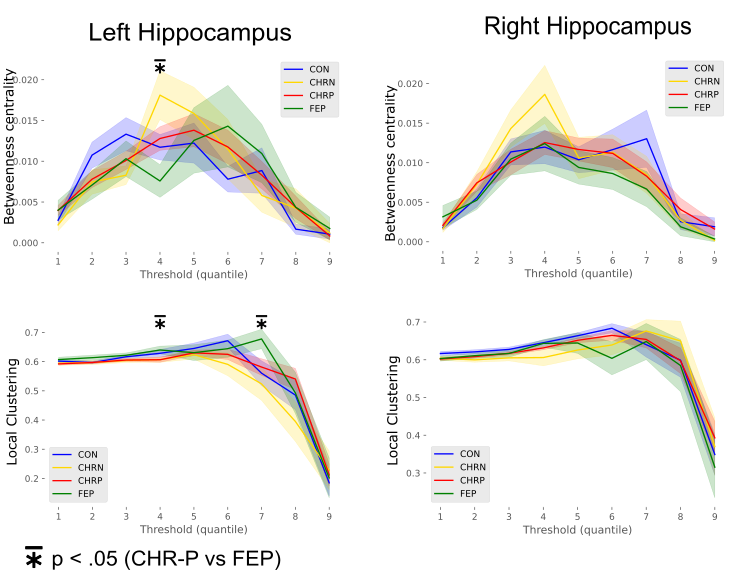


**Supp. Figure 2.**: Betweenness centrality and local clustering for the left and right hippocampus. Shown are all thresholding percentiles (1-9), but statistical testing is restricted to percentiles 3-7. Highlighted (*) are significant differences between CHR-P and FEP, with the other groups not showing significant differences; shaded regions reflect one standard deviation from the mean. FEP showed significantly lower hippocampus betweenness centrality compared to CHR-P, and increased local clustering in the left hemisphere. No group differences were detected when averaging across percentiles of interest. Betweenness centrality indicates the extent to which the hippocampus is involved in information flow between different clusters within the network, and local clustering indicates the extent to which the immediate neighbours of the hippocampus are connected to each other.
